# Monstrosity

**Published:** 1840

**Authors:** 


### MONSTROSITY.

A distant correspondent, laudably desiring to appear among the *lions* of our Journal, has sent us an account of a case, in which a woman, during gestation, was so frightened by the roaring of a lion in a menagerie, that she was afterwards delivered of a "little lion, marked, as the lion was, with a sore on its back, covered with hair." We hope to make this *lusus naturæ*, the head *lion* of our Cabinet of Morbid Anatomy, and shall give a detailed anatomical and physiological description of it *as soon as it is received.* D.

